# RNA nanoparticle as a vector for targeted siRNA delivery into glioblastoma mouse model

**DOI:** 10.18632/oncotarget.3632

**Published:** 2015-03-23

**Authors:** Tae Jin Lee, Farzin Haque, Dan Shu, Ji Young Yoo, Hui Li, Robert A. Yokel, Craig Horbinski, Tae Hyong Kim, Sung-Hak Kim, Chang-Hyuk Kwon, Ichiro Nakano, Balveen Kaur, Peixuan Guo, Carlo M. Croce

**Affiliations:** ^1^ Department of Molecular Virology, Immunology and Medical Genetics, Comprehensive Cancer Center, The Ohio State University, Columbus, OH, USA; ^2^ Department of Pharmaceutical Sciences, Nanobiotechnology Center, Markey Cancer Center, College of Pharmacy, University of Kentucky, Lexington, KY, USA; ^3^ Department of Neurological Surgery, Dardinger Laboratory for Neuro-oncology and Neurosciences, The Ohio State University Medical Center, Columbus, OH, USA; ^4^ Division of Neuropathology, Department of Pathology, University of Kentucky, Lexington, KY, USA; ^5^ ProteomeTech Inc., Seoul, Korea; ^6^ Neurosciences Research Program, Aurora Health Care Inc., Milwaukee, WI, USA

**Keywords:** pRNA, nanoparticle, three-way junction, glioblastoma, siRNA

## Abstract

Systemic siRNA administration to target and treat glioblastoma, one of the most deadly cancers, requires robust and efficient delivery platform without immunogenicity. Here we report newly emerged multivalent naked RNA nanoparticle (RNP) based on pRNA 3-way-junction (3WJ) from bacteriophage phi29 to target glioblastoma cells with folate (FA) ligand and deliver siRNA for gene silencing. Systemically injected FA-pRNA-3WJ RNPs successfully targeted and delivered siRNA into brain tumor cells in mice, and efficiently reduced luciferase reporter gene expression (4-fold lower than control). The FA-pRNA-3WJ RNP also can target human patient-derived glioblastoma stem cells, thought to be responsible for tumor initiation and deadly recurrence, without accumulation in adjacent normal brain cells, nor other major internal organs. This study provides possible application of pRNA-3WJ RNP for specific delivery of therapeutics such as siRNA, microRNA and/or chemotherapeutic drugs into glioblastoma cells without inflicting collateral damage to healthy tissues.

## INTRODUCTION

The most common primary brain tumors in adults are glioblastomas, which are also one of the most deadly cancers [1]. For glioblastomas, conventional treatment involves surgical resection followed by radiation and concurrent chemotherapy. Even with this treatment regimen, the median survival of patients with glioblastoma is less than 15 months. The poor prognosis is primarily due to tumor recurrence, which is thought to originate from a subset of cancer stem cells that survive the primary treatments. Recent studies suggested that glioblastoma stem cell survived the therapeutic stresses and become more aggressive when they recur, developing resistance to the primary chemotherapy. We sought a new strategy targeting both brain tumor cells and glioblastoma stem cells to treat the primary brain tumor and prevent tumor recurrence. RNA nanotechnology has been rapidly growing as a new generation platform for biological and medical application [2, 3]. As nanotechnology rapidly evolves, many attempts have been made to deliver small interfering RNA (siRNA) using viruses, liposome, lipid, and polymer based nanoparticles [4]. For maximal potential, such nanoparticles must be delivered systemically and specifically target intracranial tumors with minimal toxicity. Recently, it was reported the pRNA three-way junction (pRNA-3WJ) of the bacteriophage phi29 DNA packaging motor can be used to fabricate a RNA nanoparticle (RNP) with precise control of shape, size and stoichiometry [4-10]. Creation of boiling resistant RNPs with controllable shapes and defined stoichiometry has been recently reported [11]. The pRNA-3WJ nanoparticles with 2′-Fluoro (2′-F) modifications of U and C nucleotides renders the RNPs resistant to RNase degradation enhancing their *in vivo* half-life while retaining authentic functions of the incorporated modules [7, 12, 13]. Furthermore, the pRNA-3WJ RNPs were non-toxic, non-immunogenic, and displayed favorable biodistribution and pharmacokinetic profiles in mice [14]. These favorable characteristics make this novel platform attractive for the systemic delivery of siRNA to glioblastoma. One promising ligand for nanoparticle therapy in glioblastoma targeting is folate, a natural member of the B-vitamin family. Folate is required for early neuronal development and differentiation [15]. Its transportation across the blood-cerebrospinal fluid barrier (BCSF) occurs by the choroid plexus [16]. The choroid plexus expresses the largest amount of folate receptor (FR) in a body, while no FR expression is detected in cerebellum, cerebrum or spinal cord [17, 18]. Since folate is an essential component required during DNA replication and methylation in highly proliferating cells, many cancer cells, such as those of the brain, ovary, lung, breast, kidney, endometrium, colon and bone marrow, over-express FRs to increase folate uptake [19]. Folic acid (FA), a synthetic oxidized form of folate, has been widely used as a ligand in various cancer targeting materials [20].

Herein, we report a new strategy to target and deliver therapeutic siRNA to brain tumors using FA-conjugated pRNA-3WJ RNP. We first established intracranial tumor xenograft models in mice and then systemically administered RNPs through the tail vein. Based on fluorescence imaging, we demonstrated that the pRNA-3WJ RNP efficiently targeted and internalized into brain tumor cells through FR-mediated endocytosis with little or no accumulation in adjacent healthy brain cells. Gene silencing by the RNPs was also demonstrated within the luciferase gene expressing brain tumors. More importantly, pRNA-3WJ RNPs were also capable of targeting brain tumor stem cells derived from a human patient. The data demonstrate that artificially engineered RNPs can specifically target brain tumor cells, including glioblastoma stem cells, and deliver functional siRNA and possibly even therapeutic microRNAs (miRNAs) [21]. The results will pave the way for developing multifunctional RNPs for disrupting pathways involved in glioblastoma biology.

## RESULTS

**Figure 1:**
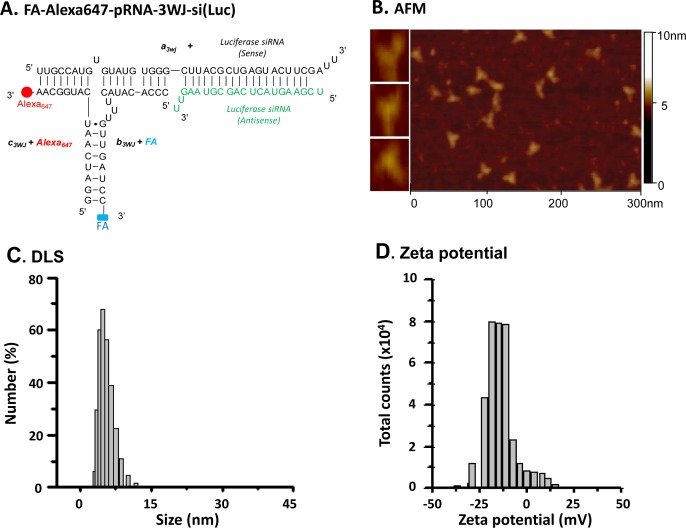
Construction and characterization of multi-functional pRNA-3WJ RNP for glioblastoma cell targeting **A**, Construction map of trivalent FA-Alexa647-pRNA-3WJ-si(Luc) RNP harboring three functionalities: Folate (FA) as a targeting ligand; Alexa647 as an imaging module; and luciferase siRNA for gene silencing. **B**, Atomic force microscopy (AFM) image showing three-branched triangular structure of self-assembled trivalent FA-pRNA-3WJ-si(Luc) RNP. **C**, Dynamic light scattering (DLS) data showing the size of FA-pRNA-3WJ-si(Luc) RNP. D, Zeta potential of FA-pRNA-3WJ-si(Luc) RNP. The data in C and D were obtained from three independent experiments.

This study was to assess application of pRNA-3WJ RNP for systemic delivery of therapeutic RNA, such as siRNA and miRNA, into brain tumors in a mouse model system. For targeted delivery of siRNA into brain tumors, a multifunctional RNP was constructed, as previously described [7, 12, 13, 22], using a scaffold based on pRNA sequences of phi29 bacteriophage with slight modifications (see *Materials and Methods*). Three RNA modules individually transcribed *in vitro* or synthesized chemically were mixed at equal molar ratio and formed three-branched RNP *via* one-step self-assembly. Each RNA module was designed to carry a functional moiety: 1) FA as the FR targeting ligand; 2) fluorophore Alexa647 as the imaging agent; and 3) luciferase siRNA as the gene silencing functional moiety or scrambled RNA as a negative control (Fig. 1A) [7, 12, 13, 22]. The resulting RNP was named FA-pRNA-3WJ-si(luc) RNP. Observation of the self-assembled FA-pRNA-3WJ-si(luc) RNP under atomic force microscopy (AFM) revealed the formation of homogeneous three-branched architectures with 3WJ core in the center (Fig. 1B), confirming the previous reports that modifications on each RNA module did not abrogate the shape-controlled self-assembly to retain the pRNA-3WJ core structure essential for homogeneous RNP formation. Dynamic light scattering (DLS) determined average hydrodynamic diameters of FA-pRNA-3WJ-si(luc) RNP to be 5.2 ± 1.2 nm (Fig. 1C), which was smaller than the predicted size (10 × 4 × 2 nm) calculated by RNA folding software based on expected duplex helix parameters and base pair lengths of the three individual RNA modules. The discrepancy between DLS measurement and computational prediction implies that each protruded branch of FA-pRNA-3WJ-si(luc) RNP was avoided from averaging three dimensions due to rapid motion of RNPs in solution. Another factor that needs to be addressed for successful systemic *in vivo* application of nanoparticles is freedom from aggregation to avoid rapid clearance from the body and diminished specific interaction between the conjugated ligand and cellular target receptors. Aggregation depends largely on the surface charge of nanoparticles and the surface of RNA is indeed highly charged. Aggregation will also change the surface charge proportional to the extent of size increase. To determine the aggregation extent, FA-pRNA-3WJ-si(luc) RNP was subjected to zeta potential analysis to measure the particle surface charge. Zeta potential of FA-pRNA-3WJ-si(luc) RNP in DEPC H_2_O was measured as a single peak at −15.8 ± 5.6 mV (Fig. 1D), indicating that most FA-pRNA-3WJ-si(luc) RNP exist as a single form without aggregation. These physical properties favor the FA-pRNA-3WJ-si(luc) RNP for systemic *in vivo* application.

**Figure 2:**
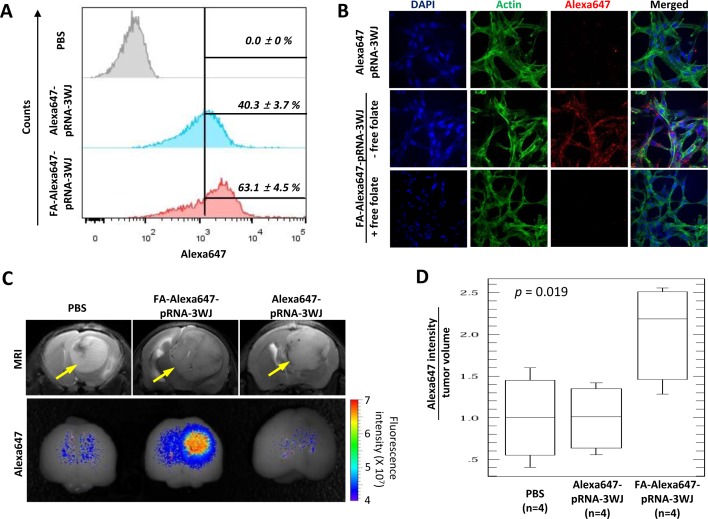
FA-mediated human glioblastoma cell targeting by FA-Alexa647-pRNA-3WJ RNP *in vitro* and *in vivo* **A**, Flow cytometry analysis for FA-dependent human glioblastoma cell U87EGFRvIII targeting *in vitro* by FA-Alexa647-pRNA-3WJ RNP. Alexa647 signals from U87EGFRvIII cells treated with 200 nM of FA-Alexa647-pRNA-3WJ RNP were compared to control RNP (FA-free Alexa647-pRNA-3WJ RNP) normalized to PBS control. Percentage of cell populations were analyzed by student *t*-test (*p* < 0.001, n=4). **B**, Immunofluorescence confocal microscopy for FA-dependent human glioblastoma cell U87EGFRvIII targeting *in vitro* by FA-Alexa647-pRNA-3WJ RNP (middle) in comparison to control RNP (FA-free Alexa647-pRNA-3WJ) (top) or 1 mM free folate pre-treated cells in culture media (bottom). Pseudocolor was used for nucleus (blue), cytoskeleton (green) and Alexa647 (red). **C**, U87EGFRvIII-induced brain tumors in mice targeted by FA-Alexa647-pRNA-3WJ RNP. Tumors were determined by MRI (yellow arrows in top panel) and visualized by fluorescence *in vivo* imaging (bottom panel) after tail vein injection of FA or FA-free Alexa647-pRNA-3WJ RNP. Representative images from each group of 4 were displayed. **D**, ANOVA analysis on fluorescence intensity of Alexa647 normalized by tumor volume (mm^3^), *p*=0.019 (n=4).

Human glioblastoma cells are known to overexpress FR, while normal brain cells show no FR expression [17-19]. To determine the specific recognition and binding capability of FA-pRNA-3WJ-si(luc) RNP towards human glioblastoma cells, firstly association of FA-Alexa647-pRNA-3WJ with U87EGFRvIII cell was tested *in vitro* in comparison to FA-free control RNP (Alexa647-pRNA-3WJ). Flow cytometry analysis showed a higher association of FA-Alexa647-pRNA-3WJ with U87EGFRvIII cells (63.1 ± 4.5%) than that of Alexa647-pRNA-3WJ (40.3 ± 3.7%) (student *t*-test, *p* < 0.001, n=4) (Fig. 2A). When FRs of U87EGFRvIII cells were pre-masked by incubating with 1 mM free-folate for 1 hr of culture before the RNP binding, the association between FA-Alexa647-pRNA-3WJ and U87EGFRvIII cells was decreased to an extent similar to the negative control Alexa647-pRNA-3WJ (Supplementary Fig. S1), indicating that the association between FA-Alexa647-pRNA-3WJ and U87EGFRvIII cells was FR dependent. The FR-mediated specific binding of FA-Alexa647-pRNA-3WJ to U87EGFRvIII cells was further confirmed by visualizing the Alexa647 signal from surface-cultured U87EGFRvIII cells treated with FA-Alexa647-pRNA-3WJ RNP under confocal fluorescence microscope. Higher fluorescence intensity of Alexa647 dye was observed from U87EGFRvIII cells treated with FA-Alexa647-pRNA-3WJ than those with control RNP (Alexa647-pRNA-3WJ) (Fig. 2B). Again, the FA-dependent association of FA-Alexa647-pRNA-3WJ RNP was abolished by pre-treatment of U87EGFRvIII cells with 1 mM free folate in culture medium (Fig. 2B). The FR-mediated specific association between FA-Alexa647-pRNA-3WJ RNP and human glioblastoma cell was also observed with other glioblastoma cell lines including T98G (Supplementary Fig. S2). Taken together, FA-conjugated pRNA-3WJ RNP has the capability to recognize and bind to human brain tumor cells through FR.

Next, we tested whether FA-pRNA-3WJ RNP can specifically target tumor cells *in vivo* using an orthotropic mouse model of glioblastoma. On the 14th day post U87EGFRvIII cell implantation into nude mouse brain, intracranial tumor growth in mice was determined by MRI (Fig. 2C, top) and randomly separated into three groups for injection of PBS and Alexa647-pRNA-3WJ as two negative controls, and FA-Alexa647-pRNA-3WJ as experimental. Each group of mice (n=4) was injected *via* tail vein (1 mg/kg of RNP in 100 μL of PBS). Fifteen hours post injection, the mice brains were dissected and subjected to fluorescence imaging to detect the Alexa647 signal from RNP. A higher fluorescence signal of Alexa647 was observed in the brains of mice injected with FA-Alexa647-pRNA-3WJ than that in the mice brains injected with control RNP (Alexa647-pRNA-3WJ) (Fig. 2C). ANOVA analysis on the fluorescence intensity from each group (n=4) normalized by their tumor volumes (Alexa647 intensity/tumor volume) confirmed the significant increase in average fluorescence intensity in the mouse brains treated with FA-Alexa647-pRNA-3WJ (2.052±0.416, s.e.m.) compared to Alexa647-pRNA-3WJ (1.014±0.279, s.e.m.) (*p*=0.019) with respect to PBS (1.000±0.298, s.e.m.) (Fig. 2D). The brain tumor region was frozen sectioned (10 μm thick) and further examined under a fluorescence confocal microscope. It revealed that FA-Alexa647-pRNA-3WJ RNP was mostly associated with counterstained brain tumor cells (Supplementary Fig. S3). These *in vivo* data strongly indicated that systemically injected FA-Alexa647-pRNA-3WJ RNP can travel to brain tissue, and successfully recognize and bind human glioblastoma cells through FA-FR interaction, rather than randomly distribute throughout the entire brain tissues.

**Figure 3:**
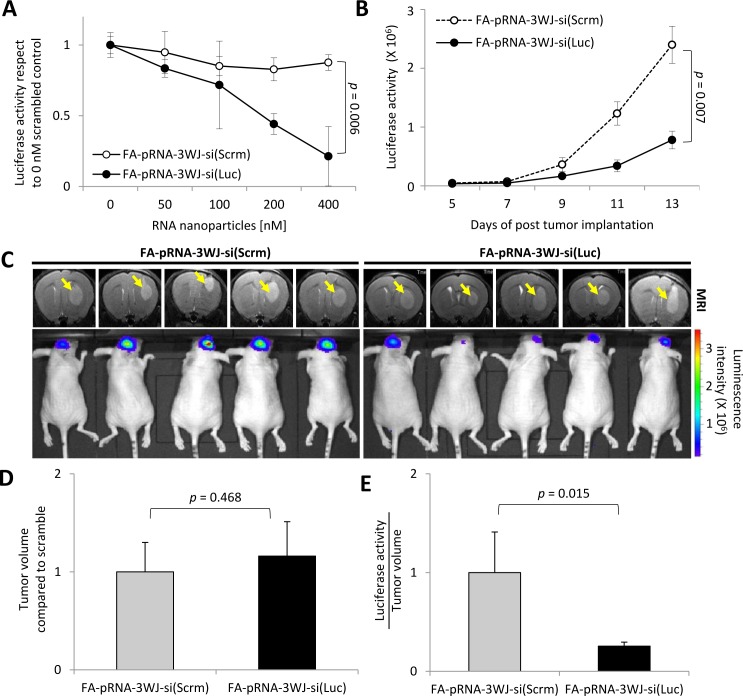
Gene silencing effect of FA-pRNA-3WJ-si(Luc) RNP in human glioblastoma cells and derived tumor **A**, A wide range (up to 400nM) of FA-pRNA-3WJ-si(Luc) (closed circles) or FA-pRNA-3WJ-si(Scrm) (negative control, open circles) RNPs were incubated with U87EGFRvIII-Luc cells *in vitro* (n=4). The change of luciferase activity was monitored *versus* the concentration of the RNPs. **B**, Luciferase gene silencing effect of FA-pRNA-3WJ-si(Luc) *in vivo* after total of three injections. Luciferase activity change by FA-pRNA-3WJ-si(Luc) (closed circles) or FA-pRNA-3WJ-si(Scrm) (open circles) were compared by mean bioluminescence intensity (n=5), *p*=0.007. **C**, Representative *in vivo* MRI images for tumor volume and bioluminescence intensity for luciferase activity from both FA-pRNA-3WJ-siRNA(Luc) or FA-pRNA-3WJ-si(Scrm) after three injections. **D**, Tumor volumes calculated from MRI compared to scrambled control group at day 13 post-xenograft, *p*=0.468 (n=5). **E**, Mean fluorescence intensity divided by tumor volume (mm^3^) was used to normalize the variation among the tested mice, *p*=0.015 (n=5). All error bars indicate s.e.m., and student *t*-test was used for statistical analysis.

After binding to target glioblastoma cells, RNP needs to internalize to deliver its cargo, siRNA, for successful target gene silencing, which is the most critical property for any nanoparticle to claim its therapeutic application. In order to test whether siRNA-loaded FA-3WJ RNP can silence the target gene in glioblastoma cells in mouse brain after systemic administration, we set up a luciferase-based gene expression reporter system by implanting luciferase gene-expressing U87EGFRvIII cells (U87EGFRvIII-Luc) in mouse brain. For a preliminary *in vitro* test, U87EGFRvIII-Luc cells were incubated for 72 hrs in culture medium containing a range between 0 and 400 nM of FA-pRNA-3WJ-si(Luc) or scrambled RNA-conjugated control FA-pRNA-3WJ-si(Scrm) RNPs without any transfection agent. After 72 hrs, FA-pRNA-3WJ-si(Luc) clearly reduced luciferase activity in a concentration dependent manner. At 400 nM, average luciferase activity in U87EGFRvIII-Luc cells incubated with FA-pRNA-3WJ-si(Luc) was decreased about five folds (0.214±0.210, s.e.m.) with respect to 0 nM. However, FA-pRNA-3WJ-si(Scrm) did not significantly reduce luciferase activity in the cells (0.876±0.056, s.e.m.) compared to 0 nM. The difference of luciferase activity at 400 nM between FA-pRNA-3WJ-si(Luc) and FA-pRNA-3WJ-si(Scrm) was statistically significant (*p*=0.006) (Fig. 3A). For *in vivo* test, intracranial tumor in mice was induced by implanting U87EGFRvIII-Luc cells. Bioluminescence signal measured from the resulted brain tumor is expected to correlate with tumor growth. When a group of brain tumor-bearing mice (n=5) were systemically injected with FA-pRNA-3WJ-si(Scrm) (1 mg/kg in 100 μL of PBS) for a total of three times over 6 days, the luciferase activity rapidly increased as the tumor grew indicating no effect of the control RNP on luciferase gene expression. However, luciferase activity from the group of mice (n=5) injected with FA-pRNA-3WJ-si(Luc) was observed to increase very slowly over time (Fig. 3B). After 3 injections, the luciferase activity from the mice injected with FA-pRNA-3WJ-si(Luc) was significantly lower (*p*=0.007) than that from the control group mice injected with FA-pRNA-3WJ-si(Scrm). The luciferase activity from the tested mice at day 13 post tumor implantation was mostly lower than that from the mice treated with FA-pRNA-3WJ-si(Scrm) (Fig. 3C). However, MRI revealed that the relative tumor size between those two groups was statistically insignificant (1.160±0.352 mm^3^, s.e.m. with respect to 1.000±0.300 mm^3^ in negative control group) (*p*=0.468, n=5) (Fig. 3D). When their luciferase activity was normalized by the tumor volumes, the relatively averaged luciferase activity over tumor volumes from the mice treated with FA-pRNA-3WJ-si(Luc) (0.255±0.040 Luminescence Radiance [p/s/cm²;/sr]/tumor volume [mm^3^], s.e.m.) was significantly lower compared to the control mice group treated with FA-pRNA-3WJ-si(Scrm) (1.000±0.410 Luminescence Radiance [p/s/cm²;/sr]/tumor volume [mm^3^], s.e.m.) (*p*=0.015, n=5) (Fig. 3E). These data strongly indicated that FA-pRNA-3WJ RNP not only specifically targeted glioblastoma cells, but also successfully internalized into the cells and delivered the cargo siRNA. The delivered siRNA, more importantly, remained functionally intact for the whole time of systemic delivery, confirming both stability and therapeutic efficacy of the FA-pRNA-3WJ RNPs. The data successfully demonstrated the therapeutic usability as a siRNA delivery system for glioblastoma treatment.

**Figure 4:**
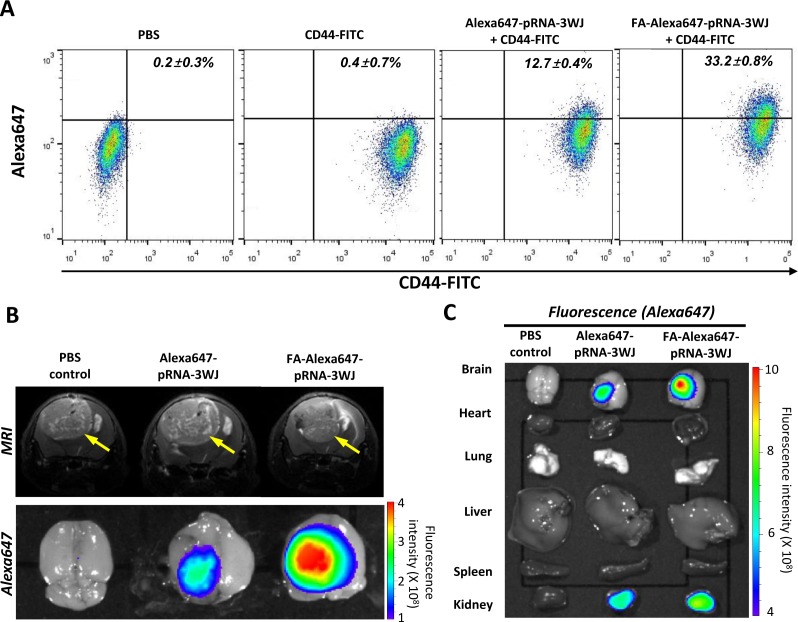
FA-mediated targeting of human glioblastoma patient-derived stem cell and derived brain tumor by FA-Alexa647-pRNA-3WJ RNPs in animal trials and biodistribution study **A**, Flow cytometry analysis for *in vitro* targeting of human glioblastoma patient-derived stem cell, 1123, by FA-Alexa647-pRNA-3WJ or Alexa647-pRNA-3WJ RNP co-treated with CD44-FITC antibody. PBS and CD44-FITC treated cells were used as gating controls. **B**, Mouse brain tumor derived from 1123 cells was evaluated by MRI for tumor size determination (top). After systemic administration of FA-Alexa647-pRNA-3WJ RNP, FA-dependent targeting was visualized by fluorescence *in vivo* imaging in comparison to FA-free Alexa647-pRNA-3WJ RNP. **C**, Biodistribution profile of FA-Alexa647-pRNA-3WJ RNP was obtained by fluorescence imaging of Alexa647 from major internal organs collected together with brain.

In clinical settings, glioblastomas are notorious for their frequent recurrence with increased aggressiveness after initial therapy, resulting in poor survival rates. It has been hypothesized that glioblastoma stem cells tend to survive the initial treatment and induce tumor recurrence, meaning that any therapeutic strategy lacking the ability to kill glioblastoma stem cells would not prevent recurrences [23]. The potential of FA-pRNA-3WJ RNPs to target glioblastoma stem cells and their derived tumor cells was investigated. We used human glioblastoma patient–derived primary neurosphere cells, named “1123”, which has been shown to possess stem cell-like characteristics including a high level of CD44 expression, self-renewal capability and tumorigenicity when implanted in mouse brain [24-26]. First, the CD44^+^ 1123 cells, maintained in serum-free sphere culture medium, were incubated *in vitro* with 200 nM of either FA-Alexa647-pRNA-3WJ or Alexa647-pRNA-3WJ RNPs. Flow cytometry analysis revealed higher FA-Alexa647-pRNA-3WJ binding to the 1123 cells than control RNP (Alexa647-pRNA-3WJ) (Fig. 4A). Compared to PBS-treated 1123 cells, 33.2 ± 0.8% of CD44^+^ 1123 cells were positively associated with FA-Alexa647-pRNA-3WJ RNP. However, Alexa647-pRNA-3WJ control RNP was associated with only 12.7 ± 0.4% of CD44^+^ 1123 cells. The difference between FA-Alexa647-pRNA-3WJ and Alexa647-pRNA-3WJ was statistically significant (*p*<0.0001).

For systemic assessment, a group of mice was then implanted with 1123 cells to induce intracranial brain tumor. Determined by MRI, mice bearing a similar size of brain tumors were then injected with PBS, Alexa647-pRNA-3WJ or FA-Alexa647-pRNA-3WJ RNPs in 100 μL of PBS through the tail vein. Fifteen hours post injection, the brains were dissected out and subjected to fluorescence imaging. Higher accumulation of FA-Alexa647-pRNA-3WJ RNP was observed in the tumor region, while less accumulation of Alexa647 signal was observed from the brains treated with either control RNP (Alexa647-pRNA-3WJ) or PBS (Fig. 4B). When two different dosages of FA-Alexa647-pRNA-3WJ RNPs (20 or 100 μg/mouse) were tested in a group of mice bearing small sized tumors, fluorescence signals were proportional to the amount of RNPs injected (Supplementary Fig. S4). These observations suggests that FA-Alexa647-pRNA-3WJ RNP can also recognize and target human glioblastoma stem cells and their derived tumor cells through FA-FR specific interaction. Throughout these studies, a fluorescence signal from the groups treated with FA-free 3WJ-pRNA control RNP was also observed although the extents were always lower than the groups treated with FA-3WJ-pRNA RNP. This might be explained by the nature of tumor induced from human patient derived stem cell-like glioblastoma cells, in which the aggressive hypervasculature leaves a large portion of blood vessels as “leaky” as they are poorly finished before forming a tight junction of the BBB, also called the EPR (enhanced permeability and retention) effect [27].

To assess the biodistribution profile of the pRNA-3WJ RNP throughout the body after systemic administration, major internal organs, including heart, lung, liver, spleen and kidney, were also collected together with brain and subjected to fluorescence imaging. Compared to brain, no significant fluorescence signal was detected from the internal organs except kidney (Fig. 4C). The biodistribution profile of FA-pRNA-3WJ RNP after its systemic administration was consistent with the previous report [28], in that FA-conjugated drugs that failed to target tumor cells are cleared from a mouse body through the kidney, reducing the safety concern of unbound pRNA-3WJ nanoparticles circulating in the blood [7, 12-14].

## DISCUSSION

For successful clinical application of pRNA-3WJ RNP for human glioblastoma detection and treatment, it was critical to evaluate its capability to access brain tumor cells by discriminating them from adjacent normal brain cells, and to have favorable biodistribution. To address those two goals, the most critical checkpoints deciding the drugability of the pRNA-3WJ nanoparticles, we employed an orthotropic intracranial glioblastoma model system in mice. According to our observations, it was clear that FA-conjugated FA-pRNA-3WJ RNP can target human glioblastoma cells through FA-FR specific interaction-mediated endocytosis by distinguishing glioma cells from adjacent normal brain cells. A series of *in vitro* experiments indicated that such targeting *in vivo* was not obviously a result of non-specific accumulation for two reasons: 1) association of FA-pRNA-3WJ RNP with glioblastoma cells was ligand-dependent; and 2) the association was mediated through FA-FR specific interaction, since free folate pre-treatment interfered with the specific interaction between FA and FRs on the targeted cells. This suggests that FA-pRNA-3WJ RNP can target and accumulate in FR^+^ glioblastoma cells. Taken together with the fact that our brain imaging data were collected 15 hrs post injection of FA-3WJ RNA nanoparticles and the luciferase gene silencing effect was seen for days, these data suggest that FA-pRNA-3WJ RNP can survive in the body by retaining the chemical integrity of cargo siRNA until it reaches the brain. Most importantly, the therapeutic delivery by the FA-3WJ RNA nanoparticles was clearly demonstrated by targeting endogenous luciferase mRNAs as a reporter system (Fig. 3). The decreased luciferase activity observed from a group of mice injected with FA-3WJ-pRNA-si(Luc) RNP clearly answered questions towards the capability of the FA-3WJ RNP regarding: 1) specific targeting to brain tumor cells; 2) internalization into brain tumor cells; and 3) releasing the functional moiety (siRNA against luciferase mRNA). In addition, the targeting capability of pRNA-3WJ nanoparticles for both brain tumor cells and glioblastoma stem cells through a FA-FR mediated manner will overcome the weak point of conventional brain tumor therapies which largely relies on surgical debulking and less-specific toxic drugs with radiation. In summary, our current study successfully demonstrated the drugability of FA-conjugated pRNA-3WJ RNP as therapeutic gene delivery for clinical applications to meet the urgent need of new strategies to target and kill both glioblastoma stem cells and their derived tumor cells. Due to the ease and flexibility of modification of each RNA module, any drug conjugation and siRNA can be loaded to the RNP as therapeutic functionalities. Recently, microRNAs have been found to involve in pathological process in glioblastoma making them as promising therapeutic targets [29-32]. Since size and working mechanism of miRNAs are similar to those of siRNAs [21], therapeutic miRNAs also can be considered to be loaded onto the pRNA-3WJ-RNP.

## MATERIALS AND METHODS

### Construction of FA-Alexa647-pRNA-3WJ-si(Luc) RNP

Multifunctional pRNA-3WJ RNP was prepared as previously described [7, 12, 13, 22] with slight modifications. In brief, three RNA modules, named a_3WJ_ (5′-UUGCCAUGUGUAUGUGGG-3′), b_3WJ_ (5′-CCCACAUACUUUGUUGAUCC-3′), and c_3WJ_ (5′-GGAUCAAUCAUGGCAA-3′), were transcribed *in vitro* or synthesized chemically using 2′-F modified nucleotides and purified separately to homogeneity. For the current study, each RNA module was further modified as following: module a_3WJ_ was extended with luciferase siRNA sequences (sense: 5′-CUUACGCUGAGUACUUCGAUU-3′ and anti-sense: 5′-UCGAAGUACUCAGCGUAAGUU-3′), or scrambled as negative control; module b_3WJ_ was conjugated with FA at the 3′ end; and module c_3WJ_ was conjugated with fluorophore Alexa647 (Alexa Fluor^®^ 647, Invitrogen) at the 3′ end. The three RNA modules were mixed at equal molar ratio to form one-step self-assembly. The self-assembled FA-Alexa647-pRNA-3WJ-si(Luc) RNPs were purified from 8 M urea-containing PAGE and frozen at −80ÐC after reconstituted in PBS. To obtain the designated concentration for each experiment, the RNPs were further diluted in PBS before use.

### Characterization of self-assembled FA-Alexa647-pRNA-3WJ-si(Luc) RNP

Three dimensional structure and shape of the final form of self-assembled FA-Alexa647-pRNA-3WJ-si(Luc) RNP was analyzed by atomic force microscopy (AFM) imaging as described previously [7, 12, 13, 22]. Apparent hydrodynamic sizes and zeta potential of pre-assembled FA-Alexa647-pRNA-3WJ-si(Luc) RNP (1.5 μM) in DEPC H_2_O was measured by Zetasizer nano-ZS (Malvern Instrument) at 25°C. The laser wavelength was 633 nm. The data were obtained from three independent measurements.

### Human glioblastoma cells and human patient-derived glioblastoma stem cells

Human glioblastoma cells, U87EGFRvIII and U87EGFRvIII-Luc (expressing luciferase reporter gene), were obtained from Dr. Webster Cavenee (Ludwig Cancer Institute, San Diego, CA). Both cells were maintained in DMEM/10% FBS/penicillin/streptomycin. Human glioblastoma patient-derived glioblastoma stem cell “1123” [24] was cultured in DMEM/F12 (Invitrogen) supplemented with B27 (1:50), heparin (5 mg/mL), basic FGF (bFGF) (20 ng/mL), and EGF (20 ng/mL). Growth factors (bFGF and EGF) were added twice a week.

### Intracranial human glioblastoma xenografts from human glioblastoma cells and human patient-derived glioblastoma stem cells

Six weeks old athymic female nu/nu mice (Jackson Laboratory, Bar Harbor, ME) were housed and handled in accordance with the Subcommittee on Research Animal Care of the Ohio State University guidelines approved by the Institutional Review Board. All mice were fed folate-free diet (Harlan, Indianapolis, IN) for at least two weeks before tumor implantation. Intracranial human glioblastoma xenograft tumor was induced by implanting human glioblastoma cell U87EGFRvIII or human patient-derived glioblastoma stem cells (1×10^5^ cells per mouse), as described previously [33]. Two weeks post intracranial tumor implantation, the location and size of the implanted tumors were determined by magnetic resonance imaging (MRI).

### Magnetic resonance imaging (MRI) for location and size of implanted brain tumor in mice

On the indicated day post-surgery of intracranial tumor injection, the location and size of the implanted tumors were determined by magnetic resonance imaging (MRI). Mouse was anesthetized with 2.5% isoflurane mixed with 1 L/min carbogen (95% O2 with 5% CO2), then maintained with 1% isoflurane thereafter. Maintaining core temperature using a warm water bath, imaging was performed using a Bruker Biospin 94/30 magnet (Bruker Biospin, Karlsruhe, Germany). Mice were injected with Magnevist, gadolinium-based contrast agent (Bayer Health Care Pharmaceuticals, Wayne, NJ) by an i.p. administration with 0.5 mmol/kg dose, then positioned in the magnet. T2-weighted RARE imaging was collected using a sequence (TR = 3524 ms, TE = 36 ms, rare factor = 8, navgs = 2, FOV=20×20 mm, 0.5 mm slice thickness). Region-of-interest (ROI) was manually outlined based on contrast in signal intensity between brain and tumor tissue.

### Fluorescence confocal microscopy for *in vitro* and *in vivo* RNP binding

For the *in vitro* targeting test of pRNA-3WJ RNP, 2 × 10^3^ of U87EGFRvIII (malignant human glioblastoma) cells in 200 μL were plated in Lab-TekII 8-well chamber slide (Nunc, Rochester, NY). The next day, the cells were washed with PBS and incubated with 200 nM of either FA-Alexa647-pRNA-3WJ RNP or control RNP (Alexa647-pRNA-3WJ) in 200 μL of culture media for 2 hrs at 37°C in a CO_2_ incubator. To block cellular FRs by free folate, PBS-washed cells were pre-treated with 1 mM free folate in 200 μL of culture media for 1 hr at 37°C in a CO_2_ incubator before RNP treatment. After washing with PBS, the RNP-treated cells were fixed in 4% paraformaldehyde (PFA) solution for 2 hrs at 4°C. The cytoskeleton of the fixed cells was stained by Alexa Fluor 488 Phalloidin (Invitrogen, Grand Island, NY) for 30 min at room temperature and the nucleus stained with 0.01% DAPI solution for 10 min at room temperature. The cells were then rinsed with PBS for 3 × 10 min and mounted with PermaFluor Aqueous Mounting Medium (Thermo Scientific). Fluorescence microscopy was performed using Olympus 4-filter-based FluoView FV1000-Filter Confocal Microscope System (Olympus Corp.) at the wavelengths of 461 nm (for the cell nucleus stained by DAPI), 530 nm (for the cytoskeleton stained by Alexa Fluor 488 Phalloidin) and 665 nm (for the Alexa647). Images were analyzed by Olympus FluoView Viewer software ver. 4.0 (Olympus). For *in vivo* targeting, the brain tumor xenograft collected 15 hrs after RNP injection was fixed in 4% PFA with 10% sucrose in PBS overnight at 4ºC and embedded in Tissue-Tek® O.C.T. compound (Sakura Finetek USA, Inc., Torrance, CA) for frozen sectioning (10 μm thick). The sectioned samples were mounted by ProLong® Gold Antifade Reagent with DAPI (Life Technologies Corporation, Carlsbad, CA) overnight. The fluorescent images were obtained using FluoView FV1000-Filter Confocal Microscope System (Olympus Corp.).

### Flow cytometry for *in vitro* RNP binding

Flow cytometry analysis was performed for *in vitro* targeting by pRNA-3WJ RNP in malignant human glioblastoma (U87EGFRvIII) and glioblastoma stem cells (1123). The cells were plated in 6-well plate one day before RNP binding. After washing with PBS, the cells were incubated with 200 nM of either FA-Alexa647-pRNA-3WJ RNP or Alexa647-pRNA-3WJ RNP in 200 μL of culture media for 2 hrs at 37°C in a CO_2_ incubator. For blocking cellular FRs by free folate, the PBS-washed cells were pre-treated with 1 mM of free folate in 200 μL of culture media for 1 hr in 37°C CO_2_ incubator before RNP treatment. After washing with PBS, the cells were harvested by trypsinization and fixed in 4% PFA solution for 2 hrs at 4°C. The cells were washed with PBS for 3 times at room temperature, then subjected to Flow Cytometry analysis using BD FACS Aria-III Cell Sorter. The data were analyzed by FlowJo 7.6.1 software.

### Systemic injection of RNPs to intracranial human glioblastoma xenograft tumor bearing mice

Based on the MRI evaluation taken one day before RNP injection, a group of mice bearing similarly sized tumors at similar location was selected for systemic injection of RNPs. Designated amount of RNPs (1 mg/kg of mouse body weight) prepared in 100 μL of PBS were injected through mouse tail vein. After 15 hrs of RNP injection, the brains were dissected out and subjected to fluorescence imaging. Tumor volume calculated from MRI was also used to normalize fluorescence intensity or luciferase activity for each mouse as described below.

### Fluorescence imaging on human glioblastoma xenograft mouse brain tumor

To investigate the delivery of pRNA-3WJ RNPs *in vivo*, a brain fluorescence imaging study was performed after tail vein injection into mice bearing brain tumor. The mice were sacrificed by cervical dislocation under anesthesia 15 hrs post injection, and brains were dissected out of mice. Fluorescence signals were detected from the dissected brains using IVIS Lumina Series III Pre-clinical *In Vivo* Imaging System (Perkin Elmer, Waltham, MA) with excitation at 640 nm and emission at 660 nm for 2 min exposure. The fluorescence intensity was expressed as Mean Radiant Efficiency [p/s/cm^2^/sr] / [μW/cm^2^], then normalized by tumor volume (mm^3^). PBS injected mice were used as fluorescence negative control. Major internal organs together with brain from the harvested mice were collected and subjected to fluorescence imaging for assessment of biodistribution profile study.

### Bioluminescence whole body imaging for luciferase activity

To investigate the siRNA delivery and silencing effect of pRNA-3WJ RNPs *in vivo*, U87EGFRvIII-Luc cell-induced brain tumor was prepared into two groups of mice (n=5). At 5, 7 and 9 days post-surgery, 1 mg/kg of mouse body weight of FA-Alexa647-pRNA-3WJ-si(Luc) RNP (or siScrm as negative control) was injected through the mouse tail vein in 100 μL of PBS. After each injection, mice were subjected to bioluminescence whole body imaging to detect the endogenous luciferase expression level. Mice were injected with 75 mg/kg Luciferin (Perkin Elmer, Waltham, MA), and anesthetized. Bioluminescence from the anesthetized mice was detected by ZFOV-24 zoom lens-installed IVIS Lumina Series III Pre-clinical *In Vivo* Imaging System (Perkin Elmer, Waltham, MA). The luminescence intensity was expressed as Averaged Radiance [p/s/cm²;/sr], then normalized by tumor volume (mm^3^).

### Statistical analysis

All statistical analyses comparing groups of mice treated with test and control RNPs were performed by either ANOVA or student t-test. *p*<0.05 was considered significant.

## SUPPLEMENTARY MATERIAL FIGURES


