# Supplementary material for: RNA nanoparticle as a vector for targeted siRNA delivery into glioblastoma mouse model
Source: Oncotarget. 2015 Mar 23;6(17):14766–76. doi: 10.18632/oncotarget.3632 (PMC4558114; doi:10.18632/oncotarget.3632)
Supplement: Supplementary file 1 [file oncotarget-06-14766-s001.pdf]

RNA nanoparticle as a vector for targeted siRNA delivery into glioblastoma mouse model

Supplementary Material

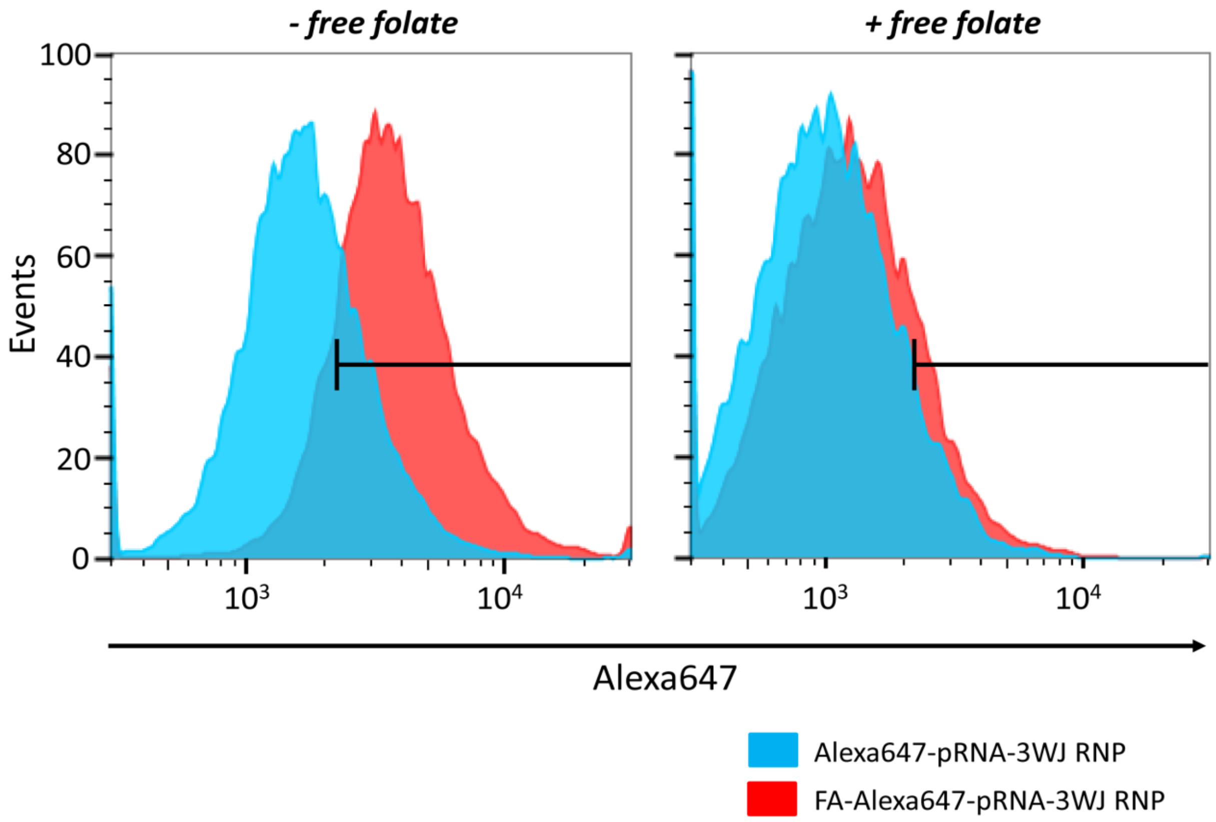

Supplementary Fig. S1: Flow cytometry analysis for FA-mediated human glioblastoma cell targeting by FA-Alexa647-pRNA-3WJ RNP *in vitro*. Human glioblastoma cells U87EGFRvIII were pre-treated with 1 mM free folate in culture media for 1 hr before incubation with FA-Alexa647-pRNA-3WJ RNP containing medium. Events as a function of Alexa647 signal intensity detected from U87EGFRvIII cells treated with 200 nM of FA-Alexa647-pRNA-3WJ RNP were compared to control RNP (FA-free Alexa647-pRNA-3WJ RNP). The figure is representative of three experiments.

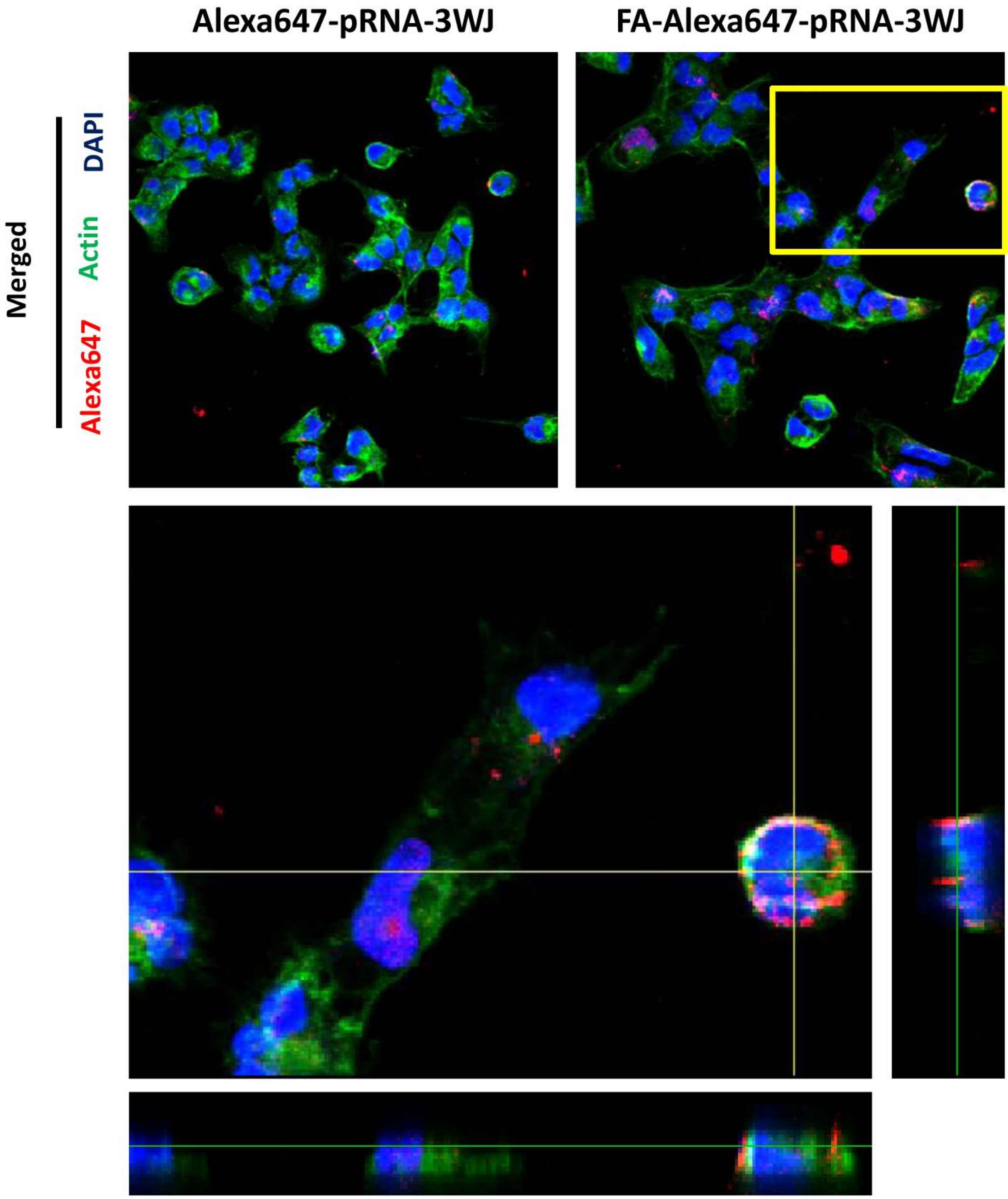

Supplementary Fig. S2: FA-mediated human glioblastoma cell T98G *in vitro* targeting by FA-Alexa647-pRNA-3WJ RNPs. Glioblastoma multiforme (GBM) cell line, T98G, was treated with 200 nM of FA-Alexa647-pRNA-3WJ or Alexa647-pRNA-3WJ for 1 hr, followed by fluorescence confocal microscopy. Pseudocolor was used for nuclear (blue), cytoskeleton (green) and Alexa647 (red).

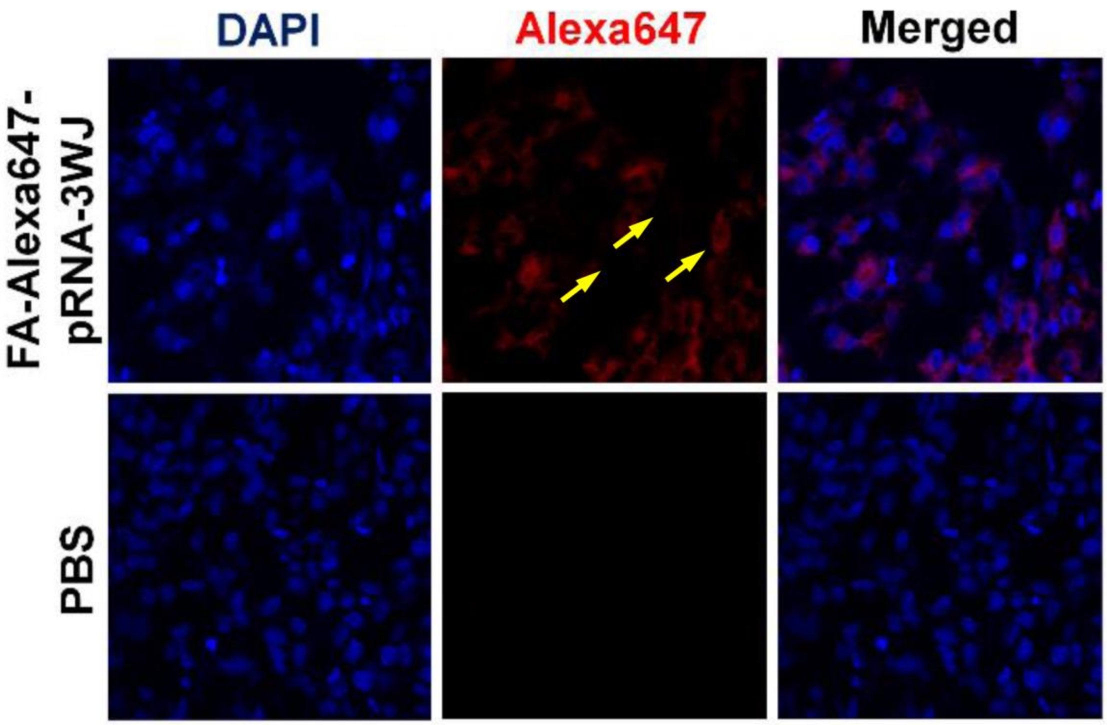

Supplementary Fig. S3: Confocal fluorescence imaging of frozen sectioned brain tumor derived from human glioblastoma patient-derived stem cell 1123 demonstrating the distribution and accumulation of FA-Alexa647-pRNA-3WJ RNP in brain tumor cells (yellow arrows). Pseudocolor was used for nuclear (blue), and Alexa647 (red).

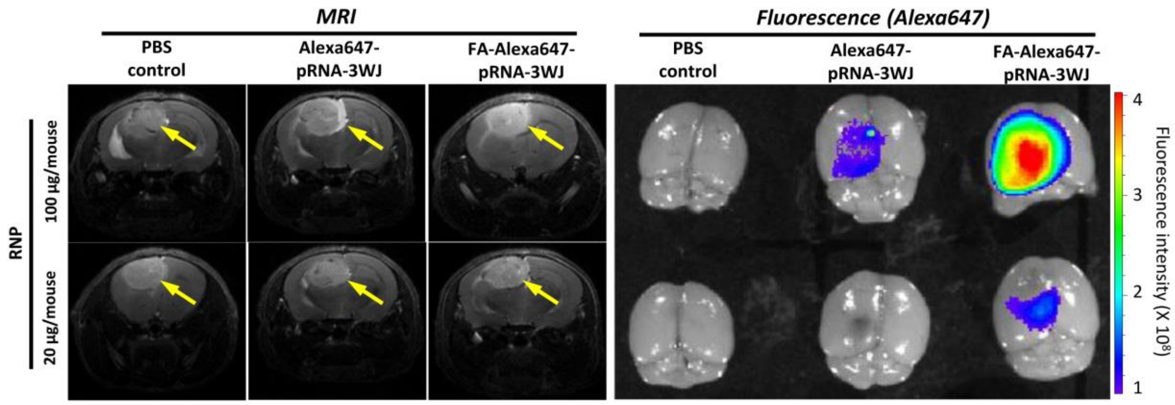

Supplementary Fig. S4: Human glioblastoma patient-derived stem cell 1123-derived mouse brain tumor targeting by FA-Alexa647-pRNA-3WJ RNPs with RNA dose-dependent (100 > 20 µg/mouse) manner. The fluorescence intensity for the tumor-bearing mouse brains were evaluated at 15 hrs after systemic injection of RNPs.
